# Interpretable machine learning prognostication of gastroenteropancreatic neuroendocrine tumors across Chinese and United States cohorts

**DOI:** 10.3389/fonc.2026.1899619

**Published:** 2026-08-13

**Authors:** Yangwei Fan, Yuqian Yang, Ke Wang, Tian Zhang, Xuyuan Dong, Yu Shi, Meichen Wang, Enxiao Li, Yinying Wu

**Affiliations:** 1 Department of Medical Oncology, The First Affiliated Hospital of Xi’an Jiaotong University, Xi’an, Shaanxi, China; 2 The Affiliated Hospital of Northwest University, Xi’an No.3 Hospital, Xi’an, Shaanxi, China; 3 School of Physics, Xi’an Jiaotong University, Xi’an, Shaanxi, China

**Keywords:** external validation, gastroenteropancreatic neuroendocrine tumors, machine learning, SHAP, survival prediction

## Abstract

**Background:**

Gastroenteropancreatic neuroendocrine tumors (GEP-NETs) display marked clinical heterogeneity, and conventional prognostic indicators such as TNM stage and Ki-67 index provide limited individualized risk discrimination. We aimed to develop and externally validate interpretable machine learning models for survival prediction.

**Methods:**

This retrospective study enrolled 337 patients with histologically confirmed GEP-NETs, randomly partitioned into a training set (n = 236) and an independent test set (n = 101) with stratification by mortality. Six survival models, including unpenalized Cox regression as a baseline, LASSO Cox, random survival forest (RSF), gradient-boosted survival analysis (GBSA), Extra Trees, and DeepSurv, were trained with 5×5 nested cross-validation. Discrimination, probabilistic accuracy, calibration, and clinical utility were comprehensively evaluated. SHAP analysis identified key predictors and informed nomogram construction. External validation was performed in 51,225 SEER patients.

**Results:**

On the test set, Extra Trees achieved the highest discrimination (C-index 0.843, 95% CI 0.762-0.914) and balanced calibration (1-year ICI 0.031). Time-dependent AUC values were 0.871, 0.847 and 0.935 at 1, 3 and 5 years. The Extra Trees-based stratification identified high-risk patients with substantially worse survival (HR 8.93, 95% CI 3.35-23.84, P<0.001). The model outperformed TNM stage (C-index 0.772), Ki-67 (0.705) and tumor grade (0.699). Decision curve analysis demonstrated greater net benefit across threshold probabilities of 5%-50%. SHAP analysis identified T stage, tumor grade, M1 status, TNM stage and Ki-67 as the top predictors. External validation in SEER preserved discriminative ability (C-index 0.719) and excellent calibration (slopes 0.901-1.058).

**Conclusion:**

Interpretable machine learning models outperform conventional approaches in predicting GEP-NET survival. Extra Trees showed the best internal discrimination and calibration, whereas DeepSurv achieved the highest external C-index. The simplified SHAP-derived nomogram provides a practical and well-calibrated but exploratory tool for individualized prognosis that requires prospective validation before clinical use.

## 1 Introduction

The incidence of gastroenteropancreatic neuroendocrine tumors (GEP-NETs) has risen substantially over the past four decades (1, 2). Data from the SEER program indicate an approximately 6.4-fold increase between 1973 and 2012 (3). These tumors exhibit considerable biological heterogeneity, ranging from indolent well-differentiated neoplasms to aggressive poorly differentiated carcinomas (3, 4). Such heterogeneity manifests across primary site, grade, stage, and treatment response, posing substantial challenges for prognostic assessment and individualized therapy (5). Accurate prognostic evaluation is essential for designing follow-up strategies, selecting adjuvant treatments, and enrolling patients in clinical trials. Predictive tools with strong discrimination, adequate calibration, and broad applicability are therefore needed.

Clinical prognostication in GEP-NETs has traditionally relied on univariate or low-dimensional indicators such as lymph node involvement, TNM stage, tumor grade, and the Ki-67 proliferation index (6, 7). Multiple independent studies have established Ki-67 as a strong prognostic marker, with its impact occasionally exceeding that of disease stage in certain settings (8). However, the TNM system is grounded in anatomic extension and cannot readily accommodate multidimensional information such as molecular pathology, which limits its utility for individualized prediction (9, 10). Recent work has emphasized that modern precision medicine requires more integrative prediction frameworks (10, 11). Against this background, nomograms based on Cox proportional hazards regression have been widely applied in GEP-NET prognostic modeling. Several SEER-based studies have developed nomograms for predicting overall or cancer-specific survival in pancreatic neuroendocrine tumors (12–14) and demonstrated superior discrimination compared with TNM staging alone. Nevertheless, these models remain constrained by the Cox framework’s reliance on a linear and proportional relationship between predictors and the log hazard ratio, which limits its ability to capture nonlinear interactions among variables (15).

Machine learning approaches have gradually emerged as a focus of cancer survival prediction owing to their advantages in handling high-dimensional data and nonlinear relationships (16, 17). Within the GEP-NET literature, a previous study built a survival prediction model using SEER liver metastasis data and reported a test C-index of 0.785 (18). Several limitations are nonetheless apparent in existing work. Most studies have relied on single-cohort validation within SEER and lack empirical evidence from Chinese populations, leaving cross-population transferability uncertain. Evaluation has often been restricted to the C-index or AUC, with limited multidimensional reporting that includes calibration performance (19). In addition, hyperparameter tuning in small cohorts has typically relied on a single hold-out set, and the resulting optimistic bias has rarely been quantified systematically.

To address these gaps, the present study compared the prognostic performance of six survival models using machine learning across a Chinese GEP-NET cohort and the SEER external cohort. Nested cross-validation was applied to mitigate overfitting, and an unpenalized Cox model served as the baseline against which several machine learning models were compared. Performance was assessed across five dimensions, namely discrimination, probabilistic accuracy, calibration, risk stratification, and net clinical benefit on decision curve analysis. To enhance clinical applicability, the optimal model was interpreted using SHAP analysis to identify the core prognostic variables, which were then used to construct a clinically operable nomogram.

## 2 Materials and methods

### 2.1 Study design and data sources

A retrospective cohort design was adopted. The internal cohort consisted of patients with histologically confirmed gastroenteropancreatic neuroendocrine tumors treated at The First Affiliated Hospital of Xi’an Jiaotong University between 2016 and 2023. The study protocol was reviewed and approved by the institutional ethics committee at The First Affiliated Hospital of Xi’an Jiaotong University (Approval No. XJTU1AF2024LSYY-101). Given the retrospective nature of the study, the requirement for written informed consent was waived. All procedures adhered to the Declaration of Helsinki.

The external validation cohort was derived from publicly released SEER records of the United States National Cancer Institute spanning 2004 to 2019. Because SEER is a public-use database, additional ethical approval was not required. Reporting of this study followed the core items of the TRIPOD (Transparent Reporting of a multivariable prediction model for Individual Prognosis Or Diagnosis) statement (20).

### 2.2 Patient inclusion and exclusion

Inclusion criteria for the internal cohort were as follows. Patients were required to have a histologically confirmed gastroenteropancreatic neuroendocrine tumor, be aged 18 years or older, and have complete clinical, pathological, and follow-up information. Only well-differentiated NETs were included. Poorly differentiated neuroendocrine carcinomas (NEC) were not eligible. Accordingly, all G3 cases corresponded to well-differentiated NET G3 under the WHO 2019 classification, and no well-differentiated and poorly differentiated G3 tumors were combined. Exclusion criteria comprised tumors arising outside the gastrointestinal tract, a history of other malignancy within the preceding 5 years, and missing values for key variables. A total of 426 patients were initially identified. After exclusion of 61 patients with incomplete follow-up and 28 patients with missing clinical variables, 337 patients were included in the analysis. Cases in which the liver was the recorded site of disease (n = 24) represented hepatic metastatic rather than primary hepatic neuroendocrine tumors. The same principles were applied to the SEER cohort. Records of neuroendocrine tumors in the gastroenteropancreatic region were retrieved using ICD-O-3 site codes. Patients younger than 18 years and those with missing survival information, incomplete staging, or unknown histologic grade were sequentially excluded, yielding 51,225 patients for external validation.

### 2.3 Candidate predictors and endpoint definition

Candidate predictors spanned four domains, namely demographic, oncologic, symptomatic, and therapeutic. Demographic variables included age and sex. Oncologic variables included primary site, TNM stage, T stage, N stage, distant metastasis status, tumor grade according to the WHO 2019 classification (21), the Ki-67 proliferation index, and functional status. Symptomatic variables comprised abdominal pain, decreased appetite, and weight loss. Therapeutic variables included radical surgery, chemotherapy, biotherapy, and targeted therapy. Comorbidities included diabetes mellitus and hypertension. The primary endpoint was overall survival, defined as the interval from the date of initial diagnosis to death from any cause or the date of last follow-up. Residual missing values in the four continuous predictors (age, Ki-67 index, T stage, N stage) were imputed with the training-set median, applied unchanged to the test set and SEER cohort. Categorical predictors were one-hot encoded with sparse categories grouped as “Other” or “Unknown”. Continuous predictors were standardized using training-set parameters. The Ki-67 index was modelled as a continuous variable throughout. As the SEER database does not record Ki-67, grade-based representative values (G1 = 3%, G2 = 10%, G3 = 40%) were assigned for the external cohort, and Ki-67 carried no information independent of grade in SEER. No pre-modeling feature-selection step was applied, with the same 46 candidate predictors entered identically into all six models (**Supplementary Figure 1**).

### 2.4 Nested cross-validation and model construction

The internal cohort was randomly partitioned into a training set (n = 236) and a test set (n = 101) at a 7:3 ratio. Stratified sampling based on death status was used, with the random seed fixed at 42 to ensure reproducibility. The test set was strictly held out from the entire modeling pipeline and was used only for final evaluation, taking no part in hyperparameter tuning or variable selection.

Because the ratio of events to covariates in the training set was relatively low, a single hold-out strategy was prone to optimistic bias during hyperparameter tuning (22). Model stability and overfitting were further evaluated by calculating the events-per-variable (EPV) ratio for the training set, comparing training- and test-set discrimination for each model, and examining the dispersion of out-of-fold C-indices from the nested cross-validation. Accordingly, 5×5 nested cross-validation was implemented on the training set. The outer 5-fold layer estimated generalization performance and the inner 5-fold layer selected hyperparameters by grid search, maximizing the mean C-index across inner folds. Six survival prediction models were compared, including the unpenalized Cox proportional hazards model, LASSO-penalized Cox regression, random survival forest (RSF) (23), gradient boosting survival analysis (GBSA), Extra Trees, and the DeepSurv deep neural network (24). For LASSO Cox, λ was selected by 10-fold cross-validation. For RSF, GBSA, and Extra Trees, the hyperparameter search covered the number of trees (fixed at 300), the minimum samples per leaf, and the number of candidate variables at each split. For DeepSurv, tuning was performed over network depth, hidden units, dropout ratio, and learning rate, using a fixed epoch budget (200 for tuning, 400 for the final model) with the lowest-loss checkpoint retained. Final hyperparameters for each model were determined by majority voting across the five outer folds. Detailed search spaces and final values are provided in **Supplementary Table 1**. All analyses used R 4.4.0.

### 2.5 Model performance evaluation

Model performance was evaluated across three dimensions, namely discrimination, probabilistic accuracy, and calibration. Discrimination was quantified by Harrell’s C-index (25). Time-dependent receiver operating characteristic analysis was used to compute the area under the curve at 1, 3, and 5 years. Probabilistic accuracy was assessed using the Brier score, and the integrated Brier score over the 0 to 60 month interval was calculated as an overall measure, with the Kaplan-Meier marginal survival function serving as the reference benchmark. Calibration was evaluated using the cloglog-transformed Cox approach to estimate the calibration slope, and the integrated calibration index along with the E50 and E90 differences were also computed. The optimal cut-off was determined on the training-set predicted scores using the maxstat method, and this threshold was then applied to the test set and the external validation cohort for risk stratification. Between-group survival differences were compared using the log-rank test. The stability of the maxstat cut-off was assessed by bootstrap resampling (B = 1000) of the training set, recomputing the optimal cut-off in each resample to obtain a 95% confidence interval. Clinical utility was assessed by decision curve analysis (26). As a literature benchmark, a Cox model using the predictor combination from a previously published SEER-based GEP-NET nomogram (27) was fit and evaluated directly on the SEER cohort. Unlike the six primary models, which were trained internally and externally validated on SEER, this benchmark reflects in-sample fit rather than external validation.

### 2.6 Model interpretability and nomogram construction

To enhance the clinical interpretability of the machine learning model, SHapley Additive exPlanations (SHAP) analysis was applied to the predictions of the optimal model (Extra Trees) on the test set (28). Global feature importance was quantified as the mean absolute SHAP value across all test-set samples. A nomogram for predicting 3-year and 5-year overall survival was then constructed using the top five original variables ranked by SHAP (29), and its calibration performance at 1, 3, and 5 years was evaluated on the test set. To facilitate clinical use, the nomogram was further deployed as a freely accessible web-based calculator, which returns individualized 3- and 5-year overall survival probabilities from these predictors. To assess the robustness of the SHAP-based feature ranking against sampling variability, permutation importance from the Extra Trees model was recomputed on 1000 bootstrap resamples of the training set.

## 3 Results

### 3.1 Cohort characteristics

A total of 337 patients with histologically confirmed gastroenteropancreatic neuroendocrine tumors were included. The pancreas was the most common primary site (n = 148, 43.9%). Tumor grade was distributed as G1 in 144 cases (42.7%), G2 in 158 cases (46.9%), and G3 in 35 cases (10.4%). Stage I and II patients accounted for 231 cases (68.6%), whereas stage III and IV patients accounted for 105 cases (31.2%). Distant metastases were present in 68 patients (20.2%), and functional tumors were observed in 59 patients (17.5%). Regarding treatment, 278 patients (82.5%) underwent resection of the primary tumor, 73 (21.7%) received biotherapy, 58 (17.2%) received chemotherapy, and 40 (11.9%) received targeted therapy. The median age was 56 years, and 154 patients (45.7%) were male.

The cohort was randomly partitioned into a training set of 236 patients and an independent test set of 101 patients at a 7:3 ratio, with stratified sampling based on death status. The two subsets were balanced with respect to age, sex, primary site, grade, stage, Ki-67 index, and major treatment exposures (**Table 1**). During the entire follow-up period, 58 deaths (17.2%) were recorded, and the median overall survival was 36.27 months. Death events numbered 41 in the training set and 17 in the test set.

**Table 1:** Baseline characteristics of the study cohort stratified by training and test sets.

Characteristic	Overall	Training	Test	p
n				
Demographic				
Age, years (median [IQR])	56.00 [47.00, 62.00]	55.50 [47.00, 62.00]	57.00 [47.00, 63.00]	0.250
Sex = Male, n (%)	154 (45.7)	109 (46.2)	45 (44.6)	0.876
Tumor pathology				
Ki-67 index (%) (median [IQR])	3.00 [2.00, 10.00]	3.00 [2.00, 10.00]	4.00 [2.00, 10.00]	0.488
T stage (median [IQR])	2.00 [1.00, 3.00]	2.00 [1.00, 3.00]	2.00 [1.00, 3.00]	0.122
N stage (median [IQR])	0.00 [0.00, 0.00]	0.00 [0.00, 0.00]	0.00 [0.00, 0.00]	0.663
M1 = Yes, n (%)	68 (20.2)	44 (18.6)	24 (23.8)	0.355
Functional tumor = Yes, n (%)	59 (17.5)	46 (19.5)	13 (12.9)	0.191
Primary site, n (%)				
Pancreas	148 (43.9)	105 (44.5)	43 (42.6)	0.567
Rectum	69 (20.5)	48 (20.3)	21 (20.8)	
Stomach	45 (13.4)	28 (11.9)	17 (16.8)	
Duodenum	42 (12.5)	28 (11.9)	14 (13.9)	
Liver	24 (7.1)	20 (8.5)	4 (4.0)	
Other	9 (2.7)	7 (3.0)	2 (2.0)	
Tumor grade, n (%)				
G1	144 (42.7)	104 (44.1)	40 (39.6)	0.526
G2	158 (46.9)	106 (44.9)	52 (51.5)	
G3	35 (10.4)	26 (11.0)	9 (8.9)	
TNM stage, n (%)				
I	129 (38.3)	93 (39.4)	36 (35.6)	0.426
II	102 (30.3)	74 (31.4)	28 (27.7)	
III	37 (11.0)	24 (10.2)	13 (12.9)	
IV	68 (20.2)	45 (19.1)	23 (22.8)	
Unknown	1 (0.3)	0 (0.0)	1 (1.0)	
Treatment, n (%)				
Surgery	278 (82.5)	198 (83.9)	80 (79.2)	0.378
Biotherapy	73 (21.7)	51 (21.6)	22 (21.8)	1.000
Chemotherapy	58 (17.2)	43 (18.2)	15 (14.9)	0.553
Targeted therapy	40 (11.9)	27 (11.4)	13 (12.9)	0.851
Comorbidities, n (%)				
Hypertension	79 (23.4)	55 (23.3)	24 (23.8)	1.000
Diabetes mellitus	30 (8.9)	24 (10.2)	6 (5.9)	0.298
Symptoms, n (%)				
Abdominal pain	123 (36.5)	92 (39.0)	31 (30.7)	0.185
Anorexia	74 (22.0)	49 (20.8)	25 (24.8)	0.505
Weight loss	70 (20.8)	46 (19.5)	24 (23.8)	0.460
Outcome				
OS, months (median [IQR])	36.27 [22.44, 53.19]	37.17 [24.22, 53.20]	31.54 [20.07, 52.99]	0.184
Death, n (%)	58 (17.2)	41 (17.4)	17 (16.8)	1.000

Continuous variables are reported as median with interquartile range (IQR), and categorical variables as count with percentage. The cohort was stratified into training and test sets at a 7:3 ratio with stratification on mortality status. IQR, interquartile range; OS, overall survival; SMD, standardised mean difference; TNM, tumor-node-metastasis.

### 3.2 Nested cross-validation

To obtain unbiased estimates of generalization performance, 5×5 nested cross-validation was performed on the training set for six survival models. These included the unpenalized Cox proportional hazards model as the reference baseline, together with five regularized or machine learning models, namely LASSO Cox, random survival forest (RSF), gradient boosting survival analysis (GBSA), Extra Trees, and DeepSurv.

The five machine learning models exhibited comparable discrimination across the outer folds, with mean C-indices clustered between 0.836 and 0.840 and stable performance across folds. In contrast, the unpenalized Cox model achieved a mean 5-fold C-index of only 0.638, which fell below the level expected from random guessing (**Supplementary Figure 2**). This finding empirically confirmed the instability of the conventional Cox model under conditions of insufficient sample-to-variable ratio and provided direct justification for the subsequent adoption of regularized and machine learning approaches. Final hyperparameters were determined through majority voting across the outer folds (**Supplementary Table 1**).

For LASSO Cox, λ was selected by 10-fold cross-validation using the minimum deviance criterion, and 20 variables with nonzero coefficients were retained. The eight variables with the largest absolute coefficients were jaundice (1.129), stage III (0.838), prior coronary heart disease (0.757), grade G1 (−0.707), M1 (0.698), local therapy (−0.535), weight loss (0.505), and stage IV (0.375). Of these, T stage, Ki-67 index, grade G3, M1, and stage IV also entered the top ten SHAP rankings of the Extra Trees model, indicating consistency between the two independent variable selection approaches on the key prognostic features (**Supplementary Table 2**).

The training set corresponded to an EPV of 0.89 (**Supplementary Table 3**). The train-test C-index gap was smaller and more consistent for the five machine-learning models (0.054–0.088) than for the unpenalized Cox model (0.122; **Supplementary Table 4**).

### 3.3 Discrimination on the test set

**Figure 1:**
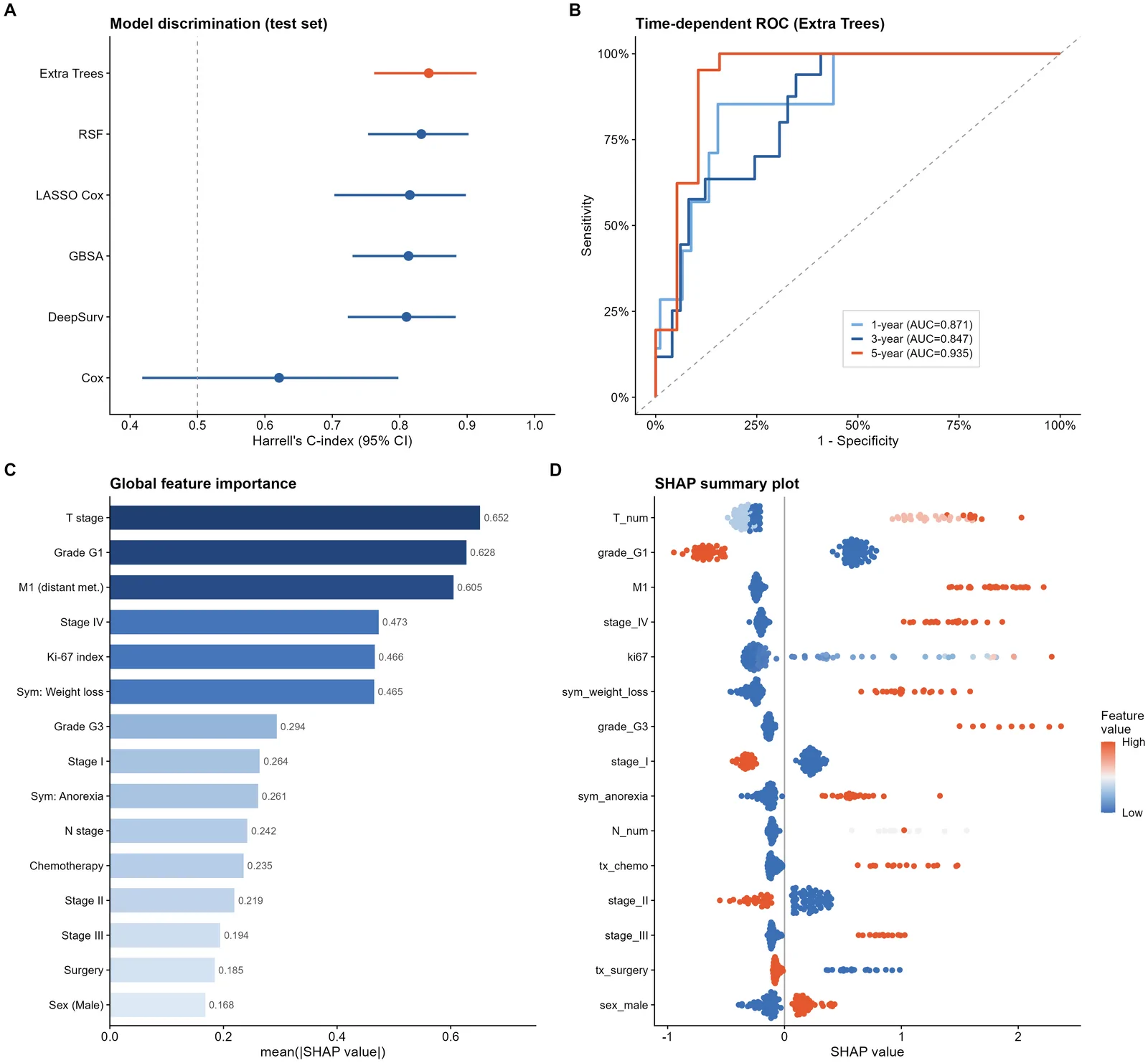
Discrimination performance and global feature importance of the survival models on the test set. **(A)** Forest plot of Harrell C-index with bootstrap 95% confidence intervals for six survival models on the test set. **(B)** Time-dependent receiver operating characteristic curves of the Extra Trees model at 1, 3 and 5 years after diagnosis, with corresponding area under the curve values. **(C)** Bar plot of the top 15 features ranked by mean absolute SHAP value derived from the Extra Trees model on the test set. **(D)** SHAP summary plot showing the distribution of SHAP values for each feature.

The six trained models were applied to the independent test set of 101 patients. Extra Trees achieved the highest discrimination, with a C-index of 0.843 (95% CI 0.762–0.914). The corresponding values for RSF, LASSO Cox, GBSA, and DeepSurv were 0.832 (95% CI 0.753–0.902), 0.815 (95% CI 0.703–0.898), 0.813 (95% CI 0.730–0.884), and 0.810 (95% CI 0.723–0.883), respectively. The unpenalized Cox model achieved a C-index of 0.621 (95% CI 0.418–0.798), which mirrored the nested cross-validation result and confirmed that it could not provide reliable prediction on these data (**Figure 1A**; **Supplementary Tables 5**, **6**).

Time-dependent ROC analysis showed that the Extra Trees model yielded 1-year, 3-year, and 5-year AUCs of 0.871, 0.847, and 0.935, respectively (**Figure 1B**). All machine learning models achieved AUCs above 0.80 at 1 and 3 years and above 0.89 at 5 years. The corresponding AUCs for the unpenalized Cox model were 0.678, 0.607, and 0.636, which remained at a low level overall.

### 3.4 Brier score and integrated Brier score on the test set

Discrimination reflects a model’s ability to rank patient risk, whereas clinical decision-making also relies on the absolute accuracy of predicted probabilities. The Brier score and integrated Brier score (IBS) were therefore used to quantify the deviation between predicted probabilities and observed events.

The Extra Trees model yielded Brier scores of 0.060, 0.110, and 0.100 at 1, 3, and 5 years, respectively, with an IBS of 0.087. The IBS values for RSF (0.089) and LASSO Cox (0.088) were comparable, and these three models constituted the lowest IBS group on the test set. The IBS values for GBSA and DeepSurv were 0.093 and 0.096, respectively, with relatively higher 5-year Brier scores of 0.118 and 0.134. The Cox model performed worst, with an IBS of 0.117 and a 5-year Brier score reaching 0.174 (**Supplementary Table 5**).

### 3.5 Calibration on the test set

Calibration was evaluated at 1, 3, and 5 years using the cloglog-Cox calibration slope and the integrated calibration index (ICI).

The Extra Trees model produced calibration slopes of 1.808, 1.671, and 1.886 across the three time points, with corresponding ICI values of 0.031, 0.031, and 0.059. The RSF model produced slopes of 2.625, 2.012, and 1.885 and ICI values of 0.036, 0.044, and 0.062. The ICI values for both tree-based models at 1 and 3 years fell below 0.05, which meets the commonly accepted clinical threshold for good calibration. LASSO Cox, GBSA, and DeepSurv yielded calibration slopes below 1 at all three time points, indicating mild divergence between the predicted and observed distributions. The Cox model showed a calibration slope close to 0, with essentially no linear association between predicted and actual survival (**Supplementary Table 6**).

Overall, all four tree-based models achieved ICI values below 0.10 at 1 and 3 years, which represents clinically acceptable calibration, and Extra Trees provided the most balanced performance across discrimination and calibration.

### 3.6 SHAP feature importance

SHAP analysis identified the top five globally important features as T stage, grade G1, distant metastasis status M1, stage IV, and Ki-67 index, with mean absolute SHAP values of 0.652, 0.628, 0.605, 0.473, and 0.466, respectively. The sixth to tenth features were weight loss (0.465), grade G3 (0.294), stage I (0.264), decreased appetite (0.261), and N stage (0.242). Chemotherapy exposure, stage II, stage III, surgical exposure, and male sex also contributed to predictions (**Figure 1C**).

The SHAP summary plot further illustrated the directional impact of each feature on the predictions (**Figure 1D**). Higher T stage, higher Ki-67 index, presence of M1, stage IV status, and the symptoms of weight loss or decreased appetite were all associated with higher predicted risk, whereas grade G1 and stage I were associated with lower predicted risk.

Bootstrap resampling (B = 1000) confirmed that the top five SHAP-ranked features (T stage, Grade G1, M1, Stage IV, Ki-67 index) were also the most stable, each ranking in the top ten in at least 89% of resamples (**Supplementary Table 7**).

### 3.7 Subgroup analysis

**Figure 2:**
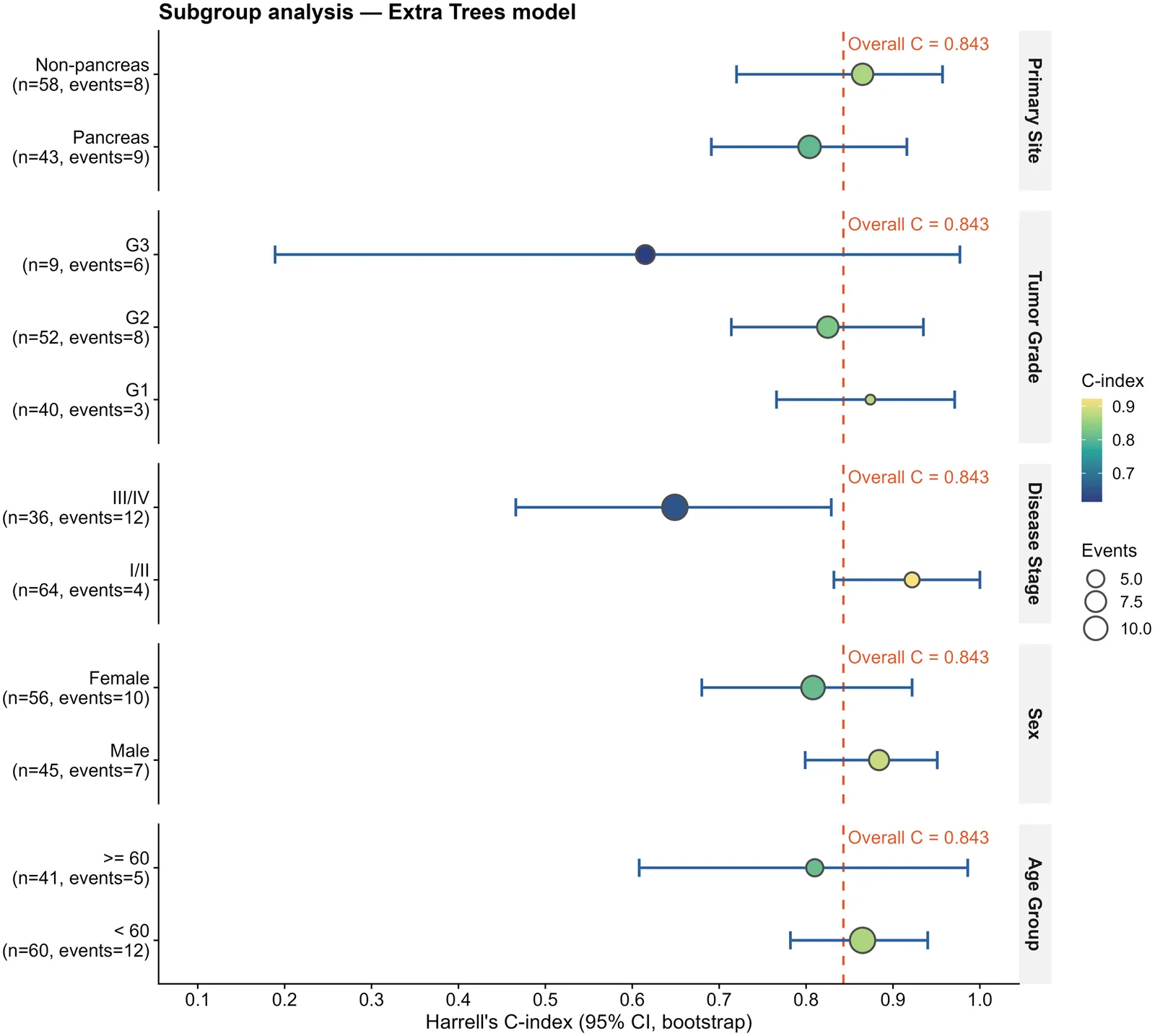
Subgroup analysis of the discriminative performance of the extra trees model on the test set.

The Extra Trees model maintained robust discrimination across multiple prespecified subgroups within the test set (**Figure 2**). Stratified by primary site, patients with pancreatic disease showed a C-index of 0.804 (95% CI 0.691–0.916), and those with non-pancreatic disease showed a C-index of 0.865 (95% CI 0.720–0.957). The C-indices for G1, G2, and G3 patients were 0.874 (95% CI 0.766–0.971), 0.825 (95% CI 0.714–0.935), and 0.615 (95% CI 0.189–0.977), respectively. Stratified by TNM stage, the combined stage I and II subgroup achieved a C-index of 0.922 (95% CI 0.832–1.000), whereas the combined stage III and IV subgroup yielded a C-index of 0.649 (95% CI 0.466–0.829). By sex, the C-index was 0.884 (95% CI 0.799–0.951) in men and 0.808 (95% CI 0.680–0.922) in women. By age, the C-indices were 0.865 (95% CI 0.782–0.940) for patients younger than 60 years and 0.810 (95% CI 0.608–0.986) for those aged 60 years or older.

### 3.8 Risk stratification and survival differences

**Figure 3:**
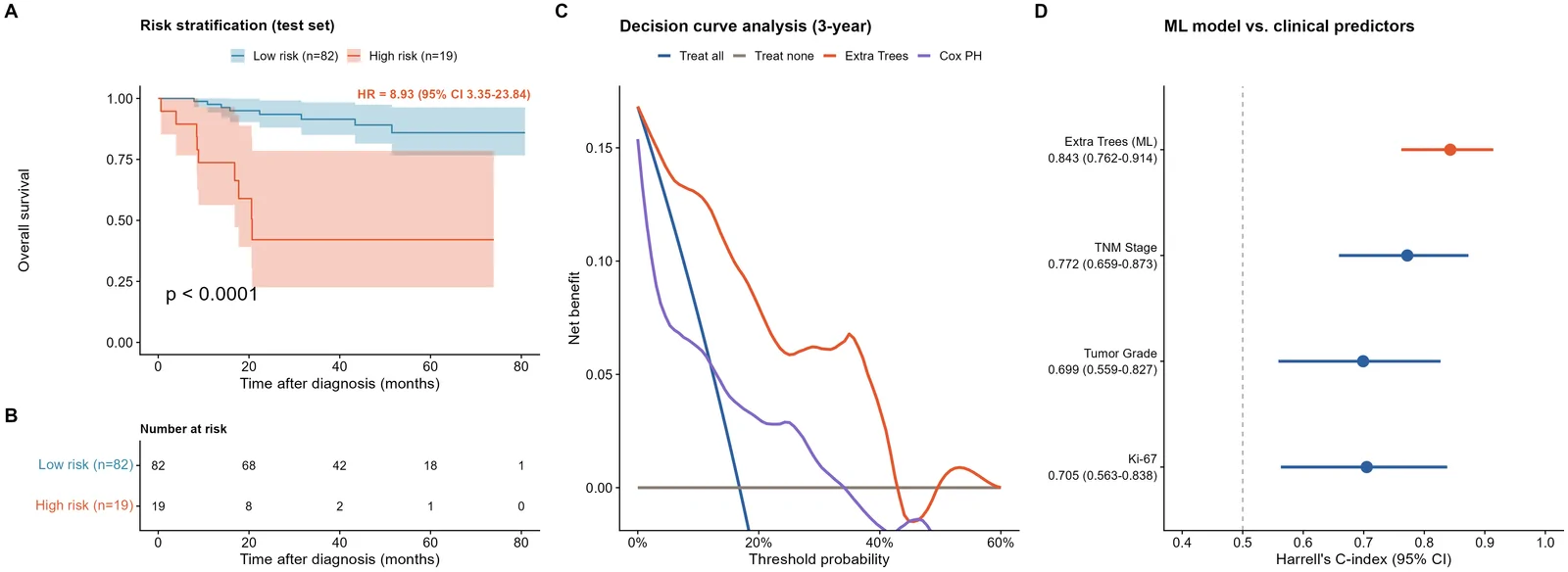
Risk stratification, decision curve analysis and comparison with conventional clinical predictors on the test set. **(A)** Kaplan-Meier curves of overall survival in the test set stratified into high-risk and low-risk groups by the optimal Extra Trees cutpoint. **(B)** Number of patients at risk in each stratum at successive time points. **(C)** Decision curve analysis at 3 years: net benefit of the Extra Trees model, unpenalized Cox model, treat-all (blue), and treat-none (brown) strategies. **(D)** Forest plot comparing the C-index of the Extra Trees model with three conventional clinical prognostic indicators on the test set.

The maxstat method applied to the Extra Trees predicted scores on the training set identified an optimal cut-off of 8.989, which assigned 82.6% of training patients to the low-risk group and 17.4% to the high-risk group (**Supplementary Table 5**). Bootstrap resampling (B = 1000) yielded a median cut-off of 8.989 (95% CI 8.227–10.909), matching the original estimate and supporting the stability of the threshold (**Supplementary Table 8**). Applying this cut-off to the test set classified 82 patients as low risk and 19 as high risk. Kaplan-Meier analysis revealed marked separation between the two survival curves, with a hazard ratio of 8.93 (95% CI 3.35–23.84) for the high-risk group relative to the low-risk group (**Figures 3A, B**). When the same approach was applied to the remaining five models, the high- and low-risk groups also showed statistically significant survival differences on the test set (**Supplementary Figure 3**).

### 3.9 Decision curve analysis and comparison with clinical prognostic indicators

Decision curve analysis at 3 years showed that, across threshold probabilities from 5% to 50%, the Extra Trees model provided greater net benefit than the Cox model and the two reference strategies of treat-all and treat-none (**Figure 3C**). Decision curve analyses at 1 and 5 years yielded consistent conclusions (**Supplementary Figure 4**).

When Extra Trees was compared with established clinical prognostic indicators on the test set, the machine learning model achieved a C-index of 0.843 (95% CI 0.762–0.914), which was substantially higher than those for TNM stage (0.772, 95% CI 0.659–0.873), Ki-67 index (0.705, 95% CI 0.563–0.838), and tumor grade (0.699, 95% CI 0.559–0.827) (**Figure 3D**; **Supplementary Table 9**).

### 3.10 Nomogram construction and calibration

**Figure 4:**
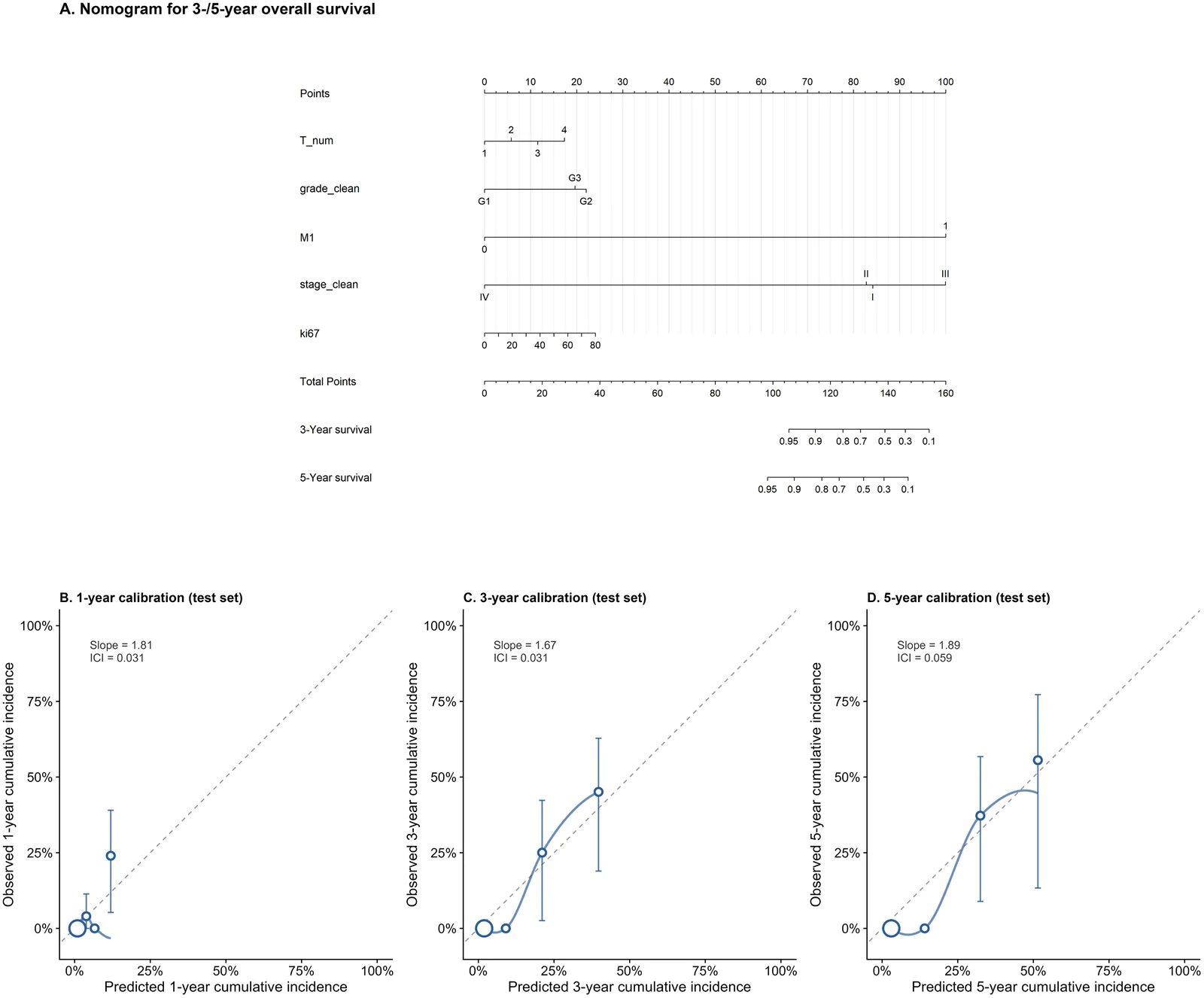
Nomogram and internal calibration of the simplified prognostic model. **(A)** Nomogram for predicting 3-year and 5-year overall survival in patients with gastroenteropancreatic neuroendocrine tumors. **(B–D)** Calibration plots of the nomogram on the test set at 1, 3 and 5 years respectively.

To provide a clinically operable prognostic tool, a nomogram for predicting 3-year and 5-year overall survival was constructed using the top five SHAP-ranked original variables, namely T stage, tumor grade, M1 status, TNM stage, and Ki-67 index (**Figure 4A**). The full nomogram formula, including all coefficients and the baseline survival function, is provided in **Supplementary Table 10**. Calibration curves on the test set at 1, 3, and 5 years showed acceptable agreement between predicted and observed cumulative incidence, although the calibration slopes (1.81, 1.67, and 1.89; ICI 0.031, 0.031, and 0.059) exceeded the ideal value of 1.0, likely reflecting the limited internal sample size and event count (**Figures 4B–D**). Calibration was close to ideal in the large external cohort. The nomogram was additionally implemented as an online survival calculator (https://nzxzds.shinyapps.io/gepnet-survival-calculator/) to support its practical application.

### 3.11 External validation in SEER

**Figure 5:**
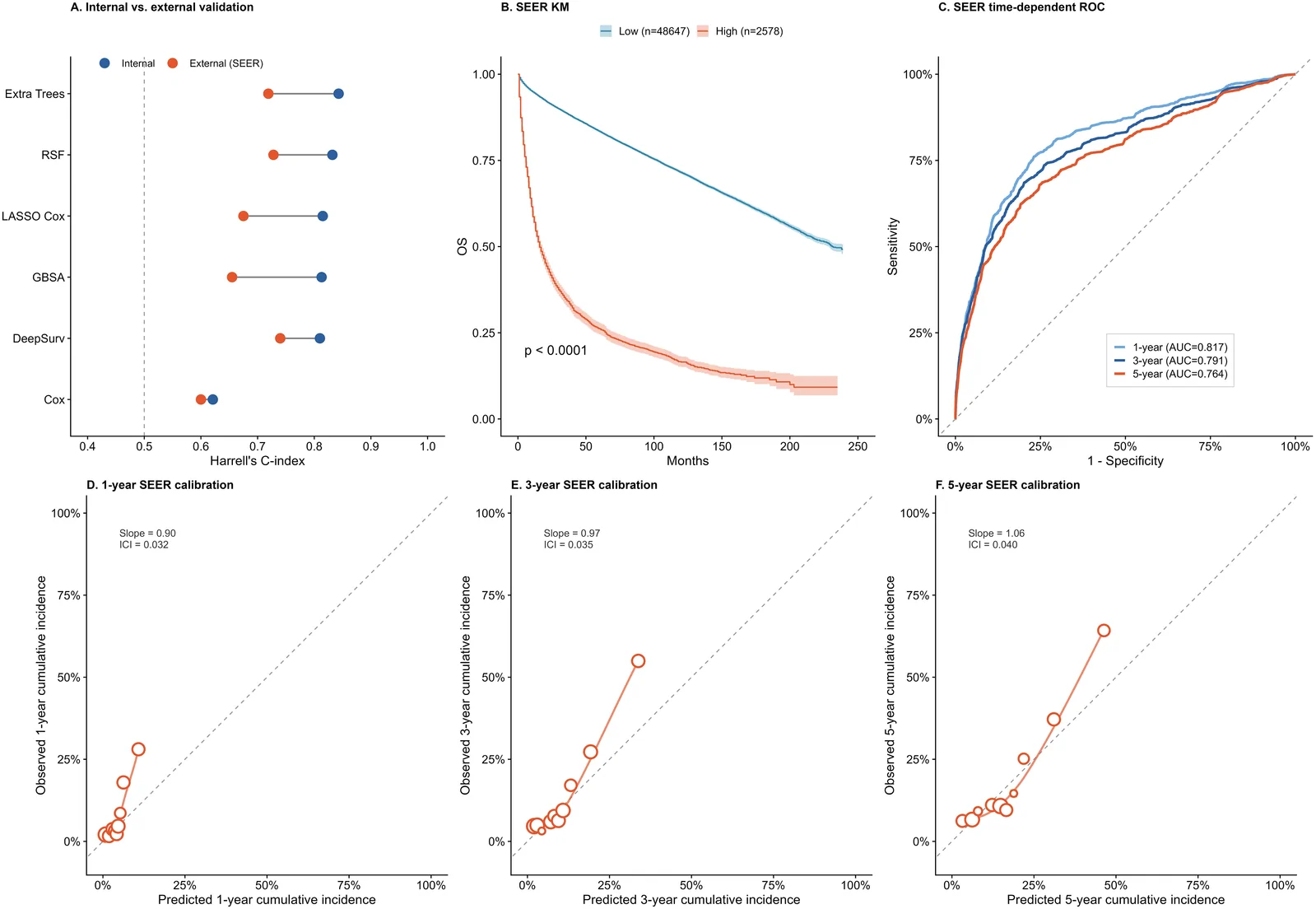
External validation of the six survival models in the SEER cohort. **(A)** Comparison of internal test-set C-index (blue) and external SEER C-index (red) for each of the six survival models. **(B)** Kaplan-Meier curves of overall survival in the SEER cohort stratified into high-risk and low-risk groups by the optimal Extra Trees cutpoint. **(C)** Time-dependent receiver operating characteristic curves of the Extra Trees model at 1, 3 and 5 years in the SEER cohort. **(D–F)** External calibration plots of the Extra Trees model at 1, 3 and 5 years respectively.

A total of 51,225 GEP-NET patients from the SEER database were included for external validation. The discrimination of each model on the SEER cohort was as follows: DeepSurv 0.740 (95% CI 0.736–0.744), RSF 0.728 (95% CI 0.724–0.732), Extra Trees 0.719 (95% CI 0.715–0.724), LASSO Cox 0.675 (95% CI 0.671–0.680), GBSA 0.655 (95% CI 0.651–0.660), and Cox 0.600 (95% CI 0.595–0.604). DeepSurv achieved the highest C-index on the external data. The 1-year, 3-year, and 5-year AUCs of Extra Trees on the external cohort were 0.817, 0.791, and 0.764, respectively (**Figure 5C**; **Supplementary Tables 11**, **12**).

All models exhibited a decrease in C-index when moving from the internal test set to the external SEER cohort (**Figure 5A**). The unpenalized Cox model showed a smaller gap between internal and external performance, but its external performance remained substantially lower than that of all machine learning models. Applying the training-derived cut-off to the SEER cohort classified 48,647 patients as low risk and 2,578 as high risk, and the survival curves of the two groups differed markedly (**Figure 5B**). This threshold was derived from the training cohort and applied without recalibration.

With respect to external calibration, the Extra Trees model achieved calibration slopes of 0.901, 0.970, and 1.058 at 1, 3, and 5 years, respectively. The corresponding ICI values were 0.032, 0.035, and 0.040, the E50 values were 0.008, 0.018, and 0.040, and the E90 values were 0.121, 0.107, and 0.080 (**Figures 5D–F**). The Extra Trees model thus maintained acceptable discrimination and good calibration in the external cohort.

The Wu et al. (27) predictor combination, fit directly on SEER, achieved a C-index of 0.809 (95% CI 0.805–0.812), higher than all six externally validated models (**Supplementary Table 7**), consistent with in-sample optimism rather than superior discrimination.

## 4 Discussion

This study developed and compared six survival prediction models in an internal cohort of 337 patients with gastroenteropancreatic neuroendocrine tumors and validated them externally in the SEER cohort. Tree-based machine learning models outperformed the unpenalized Cox regression and conventional clinical indicators across discrimination, calibration, and clinical decision utility. This advantage was not uniform across cohorts, as DeepSurv achieved the highest discrimination externally.

The unpenalized Cox model showed lower C-indices in both the internal and external cohorts, a finding consistent with the model’s instability at the present sample size. The internal training set contained only 41 death events, yielding an events-per-variable ratio well below the threshold recommended for robust modeling (30). Under such conditions, individual coefficients become highly sensitive to single events, and the model struggles to produce a stable risk ordering. After LASSO shrinkage retained 20 nonzero coefficients, the C-index rose to 0.815, which provides empirical support for this interpretation. Overfitting, EPV, model stability, and confidence in feature importance were assessed explicitly. The EPV of 0.89 falls below conventional thresholds for regression modelling (30), though this metric applies most directly to Cox regression rather than the tree ensembles or DeepSurv. Overfitting, quantified as the train-test C-index gap, was smaller and more consistent for the machine-learning models (0.054–0.088) than for Cox (0.122), consistent with the narrower dispersion of C-indices across nested cross-validation folds. Bootstrap resampling (B = 1000) further showed that the top five SHAP-ranked features were each selected within the top ten in 89–100% of resamples, whereas lower-ranked variables were markedly less stable, indicating that the core prognostic signal is robust while secondary feature rankings warrant more cautious interpretation. Calibration slopes for the two tree-ensemble models exceeded 1 at every time point. This indicates predicted risks are compressed relative to observed outcomes. The models under-discriminate at the extremes and produce conservative predictions rather than well-calibrated ones. This likely reflects the variance-shrinking effect of randomized ensembles in a small training sample. LASSO Cox, GBSA, and DeepSurv showed slopes below 1 (0.67–0.78), consistent with mild overfitting of the linear risk score. The unpenalized Cox model showed a slope near 0, reflecting essentially no linear association between predicted and observed risk. Model-specific recalibration is advisable before clinical deployment, particularly for RSF. The advantages of machine learning models can be attributed to both algorithmic and structural factors. Algorithmically, bootstrap sampling, random feature selection, and random node splitting reduce variance and yield more stable ensemble predictions in small samples. Structurally, tree-based models do not require a prespecified hazard function form and can capture the nonlinear effects of key variables. Extra Trees achieved the highest C-index on the test set, which can be attributed to its extremely-randomized splitting mechanism that further decorrelates trees and enhances robustness in small samples (31). DeepSurv displayed a different pattern from the tree-based models. Its C-index on the internal test set was 0.810 with a relatively high IBS, whereas its C-index rose to 0.740 in the SEER cohort and surpassed all tree-based models. Deep network performance is particularly sensitive to sample size, and overfitting tends to occur in small samples, whereas larger datasets allow the nonlinear representation capacity to be fully realized (32, 33). This pattern indicates that evaluation of deep survival models should jointly consider sample size and event-density stability rather than rely on a single-cohort result.

SHAP analysis identified T stage, grade G1, M1 status, stage IV, and Ki-67 as the top five variables. These results align with existing prognostic evidence for GEP-NETs, in which the importance of anatomic burden indicators such as T stage has been supported by large-scale studies. Ki-67 and tumor grade together capture proliferative activity. Although Ki-67 is partially encoded within the WHO 2019 grading scheme (21), it nevertheless entered the SHAP top five as an independent continuous variable, which suggests that it carries information beyond grade alone. This observation is consistent with recent work refining Ki-67 thresholds. One study of 190 pancreatic NET cases identified a Ki-67 value of 12% as the optimal cut-off within the G2 stratum, and patients in the G2-high and G2-low subgroups showed markedly different prognoses (34). A systematic review of 1,669 patients also suggested that continuous Ki-67 information offers incremental prognostic value (35). The tree-based models in the present study learn the nonlinear contribution of Ki-67 without prespecified cut-offs, which echoes these clinical observations. Grade G1 emerged as a strong protective variable and ranked second by SHAP, in line with prior reports that patients with Ki-67 ≤ 5% have more favorable outcomes (36). The M1 distant metastasis variable is consistent with the literature emphasizing distant metastasis, particularly hepatic metastasis, as an important prognostic factor in GEP-NETs (5, 18). Its broad SHAP distribution suggests substantial heterogeneity in the risk contribution of the M variable, in line with the previously observed heterogeneity of hepatic metastatic burden (5). Incorporation of continuous variables such as the number of liver metastases may further improve model resolution in future work. The entry of weight loss and decreased appetite into the SHAP top ten represents an important distinction between the present study and prior SEER-based work. Most SEER-based prognostic models for GEP-NETs have relied solely on demographic and tumor features, which precludes inclusion of this dimension (12, 13, 18). Mechanistically, tumor-related inflammatory cytokines and hormonal imbalance from neuroendocrine tumors jointly drive cachexia, which has been established as an independent prognostic indicator in multiple solid tumors (37). In GEP-NEN populations, weight loss and sarcopenia have been associated with shortened survival (38, 39). By incorporating subjective symptoms together with objective staging and proliferation markers, the present prediction framework more closely reflects the information available in routine clinical practice, which may partly account for the high discriminative performance of Extra Trees in this small-sample setting. In addition, LASSO Cox analysis identified jaundice as the variable with the largest weight. A 93-patient study (40) reported that jaundice was associated with high-grade PNEN and was observed exclusively in Grade 3 patients, which suggests that jaundice carries high information density as a composite indicator of tumor invasiveness and biliary obstruction. The two independent models overlapped substantially on T stage, Ki-67, Grade G3, M1, and Stage IV, which further reinforces these variables as the core signal set for GEP-NET prognosis.

Translating statistical discrimination into clinical utility represents an important extension of this work relative to previous studies (18, 41). Decision curve analysis indicated that the Extra Trees model provided quantitative support for intensified follow-up or aggressive treatment across the majority of clinically meaningful threshold ranges. The nomogram built from the top five SHAP variables achieved calibration indices below 0.06 at 1, 3, and 5 years, which enables clinicians to perform individualized prognostic estimation without dedicated software (29). As a literature benchmark, a Cox model using the Wu et al. (27) predictor set was fit directly on SEER and outperformed all externally validated models (0.809), an advantage attributable to in-sample fitting rather than genuine superiority. The externally validated estimates remain the more realistic gauge of generalizability.

Several limitations should be acknowledged. The internal cohort had a relatively small sample size and a limited number of events, which constrained the robustness of subgroup analyses. The cohort was dominated by low-grade tumors, reflecting the real-world epidemiology of GEP-NETs but limiting extrapolation to high-grade disease. Confidence intervals were widest in the smallest strata, notably Grade G3 (C-index = 0.615, 95% CI 0.189–0.977) and patients aged 60 years or older (95% CI 0.608–0.986). The lowest performance observed in the G3 subgroup should be treated as hypothesis-generating rather than robust evidence, given the wide interval and limited events. In addition, the SEER database lacks Ki-67 values and detailed treatment information, and external validation may therefore underestimate the performance attainable with complete information. The retrospective single-center design means prospective validation has not yet been performed. Clinical applicability should be confirmed prospectively before implementation. Because the risk-stratification threshold was derived entirely from the internal training cohort, external users should treat the reported cut-off as cohort-specific and consider recalibration before applying it in other populations. SHAP-based interpretation in survival settings may be influenced by anchor selection, and time-dependent approaches could provide finer-grained interpretation in future studies.

## 5 Conclusion

A machine learning prognostic framework for gastroenteropancreatic neuroendocrine tumors was developed and evaluated in an external SEER cohort. Model ranking varied by cohort size, with Extra Trees performing best internally and DeepSurv performing best externally. The framework outperformed conventional Cox regression and univariate staging indicators across discrimination and calibration in both the internal test set and the SEER cohort. A simplified nomogram built from the SHAP-derived key variables is an exploratory tool for rapid prognostic assessment, pending prospective validation. Future work should include prospective multicenter validation, integration of multimodal radiomics and molecular markers, and methodological investigation of calibration instability in deep survival models within small-sample settings.

## Data Availability

The data analyzed in this study is subject to the following licenses/restrictions: The internal cohort data analyzed in the present study are not publicly available, but they can be obtained from the corresponding author upon reasonable request and with permission from the institutional ethics committee. The external validation data were retrieved from the Surveillance, Epidemiology, and End Results (SEER) program of the United States National Cancer Institute and are publicly accessible at https://seer.cancer.gov. Requests to access these datasets should be directed to shadowless_111@163.com.
