# Supplementary material for: Interpretable machine learning prognostication of gastroenteropancreatic neuroendocrine tumors across Chinese and United States cohorts
Source: Front Oncol. 2026 Aug 13;16:1899619. doi: 10.3389/fonc.2026.1899619 (PMC13518172; doi:10.3389/fonc.2026.1899619)
Supplement: Supplementary file 1 [file Table1.docx]

***Supplementary Material***

# **1.** Supplementary Tables

**Table S1.** Performance of six survival models in 5×5 nested cross-validation on the training set.

| Model | Mean C-index | SD | Median | Min | Max | Final hyperparameters |
| --- | --- | --- | --- | --- | --- | --- |
| Extra Trees | 0.845 | 0.073 | 0.845 | 0.744 | 0.929 | mtry = 3, min.node.size = 10 |
| DeepSurv | 0.840 | 0.074 | 0.825 | 0.756 | 0.922 | lr = 1×10⁻^4^, dropout = 0.2, wd = 0.001 |
| GBSA | 0.837 | 0.049 | 0.840 | 0.775 | 0.907 | n.trees = 100, shrinkage = 0.01, depth = 2 |
| RSF | 0.836 | 0.069 | 0.838 | 0.752 | 0.929 | mtry = 3, nodesize = 20 |
| LASSO Cox | 0.836 | 0.080 | 0.851 | 0.725 | 0.933 | α = 0.5 |
| Cox | 0.638 | 0.171 | 0.640 | 0.481 | 0.892 | no tuning |

**Notes:** A 5×5 nested cross-validation was performed on the training set, with the outer 5 folds used to estimate generalization performance and the inner 5 folds used for hyperparameter selection. Final hyperparameters were determined by majority voting across the outer folds. The unpenalized Cox model was included as a reference baseline without hyperparameter tuning.

**Abbreviations:** CI, confidence interval; CV, cross-validation; GBSA, gradient boosted survival analysis; LASSO, least absolute shrinkage and selection operator; lr, learning rate; mtry, number of variables sampled at each split; RSF, random survival forest; SD, standard deviation; wd, weight decay.

**Table S2.** Variables retained by the LASSO Cox model with their corresponding regression coefficients and hazard ratios.

| **Variable** | **Coefficient** | **Hazard ratio** |
| --- | --- | --- |
| Jaundice (symptomatic) | 1.129 | 3.091 |
| Stage III | 0.838 | 2.312 |
| Coronary heart disease (history) | 0.757 | 2.133 |
| Grade G1 | −0.707 | 0.493 |
| Distant metastasis (M1) | 0.698 | 2.009 |
| Local treatment | −0.535 | 0.586 |
| Weight loss (symptomatic) | 0.505 | 1.657 |
| Stage IV | 0.375 | 1.455 |
| Liver primary | 0.289 | 1.335 |
| T stage | 0.282 | 1.326 |
| Other primary site | 0.280 | 1.322 |
| Grade G3 | 0.268 | 1.307 |
| Drinking history | −0.261 | 0.770 |
| Sex (male) | 0.207 | 1.230 |
| Hypertension (history) | −0.185 | 0.831 |
| Anorexia (symptomatic) | 0.166 | 1.181 |
| Functional tumor | −0.130 | 0.878 |
| Ki-67 index | 0.126 | 1.134 |
| Nausea/vomiting (symptomatic) | −0.061 | 0.941 |
| Immunotherapy | 0.036 | 1.037 |

**Notes:** LASSO Cox regression was fitted on the training set (n = 236) with the optimal regularization parameter λ selected by 10-fold cross-validation under the minimum partial likelihood deviance criterion. Variables are sorted by descending absolute regression coefficient. Hazard ratios are derived as exp(β).

**Abbreviations:** LASSO, least absolute shrinkage and selection operator.

**Table S3.** Events-per-variable (EPV) summary for the training set.

| **Metric** | **Value** |
| --- | --- |
| Training set N | 236 |
| Training set events | 41 |
| Candidate predictors (post one-hot encoding) | 46 |
| EPV (events / candidate predictors) | 0.89 |

**Notes:** EPV was calculated for the training set only (41 events / 46 candidate predictors after one-hot encoding). This classical heuristic was developed for regression-type survival models and is most directly relevant to the unpenalized Cox model; it does not directly account for the variance-reduction mechanisms of the ensemble and deep-learning models (bootstrap aggregation, random feature/split selection for RSF/GBSA/Extra Trees; dropout and weight decay for DeepSurv). For these models, stability was instead assessed via 5×5 nested cross-validation and the training-to-test discrimination gap.

**Abbreviations:** EPV, events per variable.

**Table S4.** Training-set versus test-set discrimination for six survival models.

| **Model** | **C-index (train)** | **C-index (test)** | **Train–test gap** | **95% CI (test)** |
| --- | --- | --- | --- | --- |
| RSF | 0.886 | 0.832 | 0.054 | 0.753–0.902 |
| DeepSurv | 0.873 | 0.810 | 0.063 | 0.723–0.883 |
| GBSA | 0.877 | 0.813 | 0.064 | 0.730–0.884 |
| LASSO Cox | 0.895 | 0.815 | 0.080 | 0.703–0.898 |
| Extra Trees | 0.931 | 0.843 | 0.088 | 0.762–0.914 |
| Cox | 0.743 | 0.621 | 0.122 | 0.418–0.798 |

**Notes:** Train–test gap is defined as C-index(train) − C-index(test) for each model, using the final hyperparameters obtained from nested cross-validation. The five machine-learning models showed comparable, modest gaps (0.054–0.088), versus 0.122 for the unpenalized Cox model, indicating that the machine-learning models did not overfit disproportionately relative to the conventional benchmark despite the limited number of training events.

**Abbreviations:** CI, confidence interval; GBSA, gradient boosted survival analysis; RSF, random survival forest.

**Table S5.** Performance of six survival models on the independent test set: Discrimination and probabilistic accuracy.

| **Model** | **C-index (95% CI)** | **AUC 1-yr** | **AUC 3-yr** | **AUC 5-yr** | **Brier 1-yr** | **Brier 3-yr** | **Brier 5-yr** | **IBS** |
| --- | --- | --- | --- | --- | --- | --- | --- | --- |
| **Extra Trees** | 0.843 (0.762–0.914) | 0.871 | 0.847 | 0.935 | 0.060 | 0.110 | 0.100 | 0.087 |
| **RSF** | 0.832 (0.753–0.902) | 0.864 | 0.832 | 0.927 | 0.060 | 0.112 | 0.103 | 0.089 |
| **LASSO Cox** | 0.815 (0.703–0.898) | 0.811 | 0.837 | 0.899 | 0.064 | 0.113 | 0.103 | 0.088 |
| **GBSA** | 0.813 (0.730–0.884) | 0.867 | 0.803 | 0.906 | 0.054 | 0.127 | 0.118 | 0.093 |
| **DeepSurv** | 0.810 (0.723–0.883) | 0.840 | 0.813 | 0.916 | 0.055 | 0.124 | 0.134 | 0.096 |
| **Cox** | 0.621 (0.418–0.798) | 0.678 | 0.607 | 0.636 | 0.065 | 0.141 | 0.174 | 0.117 |

**Abbreviations:** CI, confidence interval; GBSA, gradient boosted survival analysis; RSF, random survival forest.

**Table S6.** Performance of six survival models on the independent test set: Calibration metrics.

| **Model** | **Slope 1-yr** | **Slope 3-yr** | **Slope 5-yr** | **ICI 1-yr** | **ICI 3-yr** | **ICI 5-yr** | **E50 1-yr** | **E50 3-yr** | **E50 5-yr** | **E90 1-yr** | **E90 3-yr** | **E90 5-yr** |
| --- | --- | --- | --- | --- | --- | --- | --- | --- | --- | --- | --- | --- |
| **Extra Trees** | 1.808 | 1.671 | 1.886 | 0.031 | 0.031 | 0.059 | 0.017 | 0.025 | 0.054 | 0.092 | 0.068 | 0.116 |
| **RSF** | 2.625 | 2.012 | 1.885 | 0.036 | 0.044 | 0.062 | 0.024 | 0.041 | 0.053 | 0.100 | 0.080 | 0.130 |
| **LASSO Cox** | 0.674 | 0.674 | 0.674 | 0.047 | 0.074 | 0.075 | 0.027 | 0.026 | 0.028 | 0.110 | 0.140 | 0.119 |
| **GBSA** | 0.755 | 0.755 | 0.755 | 0.025 | 0.101 | 0.143 | 0.016 | 0.051 | 0.079 | 0.051 | 0.206 | 0.373 |
| **DeepSurv** | 0.780 | 0.780 | 0.780 | 0.034 | 0.100 | 0.148 | 0.024 | 0.055 | 0.061 | 0.072 | 0.199 | 0.373 |
| **Cox** | −0.009 | −0.009 | −0.009 | 0.026 | 0.075 | 0.097 | 0.020 | 0.085 | 0.087 | 0.068 | 0.121 | 0.192 |

**Notes:** Discrimination is quantified by Harrell C-index with bootstrap 95% confidence interval based on 1000 resamples and time-dependent area under the receiver operating characteristic curve (AUC) at 1, 3 and 5 years. Probabilistic accuracy is quantified by time-specific Brier score and integrated Brier score (IBS) over 0 to 60 months. Calibration is assessed by cloglog-Cox calibration slope and integrated calibration index (ICI), with E50 and E90 representing the 50th and 90th percentile of absolute differences between predicted and observed cumulative incidence. The ideal calibration slope equals 1; values closer to 0 indicate better calibration for ICI, E50 and E90.

**Abbreviations:** AUC, area under the curve; CI, confidence interval; GBSA, gradient boosted survival analysis; IBS, integrated Brier score; ICI, integrated calibration index; LASSO, least absolute shrinkage and selection operator; RSF, random survival forest.

**Table S7.** Bootstrap stability of Extra Trees feature importance (permutation importance, 1000 bootstrap resamples of the training set).

| **Rank** | **Feature** | **Mean importance** | **95% CI** | **Top-10 selection frequency (%)** |
| --- | --- | --- | --- | --- |
| 1 | T stage | 0.0399 | 0.0209–0.0600 | 100 |
| 2 | Grade G1 | 0.0287 | 0.0161–0.0443 | 100 |
| 3 | M1 (distant metastasis) | 0.0222 | 0.0090–0.0436 | 89 |
| 4 | Stage IV | 0.0217 | 0.0098–0.0435 | 90 |
| 5 | Ki-67 index | 0.0210 | 0.0122–0.0328 | 96 |
| 6 | Weight loss | 0.0198 | 0.0053–0.0373 | 76 |
| 7 | Chemotherapy | 0.0166 | 0.0052–0.0348 | 65 |
| 8 | Stage I | 0.0151 | 0.0062–0.0278 | 67 |
| 9 | Grade G3 | 0.0150 | 0.0022–0.0315 | 62 |
| 10 | Grade G2 | 0.0117 | 0.0048–0.0233 | 41 |
| 11 | Anorexia | 0.0114 | 0.0017–0.0258 | 43 |
| 12 | Stage II | 0.0109 | 0.0047–0.0205 | 35 |
| 13 | N stage | 0.0101 | 0.0013–0.0233 | 33 |
| 14 | Age | 0.0096 | 0.0026–0.0173 | 16 |
| 15 | Surgery | 0.0079 | 0.0014–0.0186 | 17 |

**Notes:** Each of the 1000 bootstrap resamples refit an Extra Trees model on a bootstrap sample of the training set using the final hyperparameters, and permutation importance was recorded for each candidate variable. The 95% CI reflects the 2.5th–97.5th percentile of importance across resamples; selection frequency indicates how often each variable ranked among the top ten most important features.

**Abbreviations:** CI, confidence interval.

**Table S8.** Optimal cutpoints derived from the maxstat method on the training set for risk stratification.

| **Model** | **Optimal cutpoint** | **Low risk (%)** | **High risk (%)** |
| --- | --- | --- | --- |
| **Extra Trees** | 8.989 | 82.6 | 17.4 |
| **RSF** | 8.098 | 75.4 | 24.6 |
| **GBSA** | −0.916 | 89.0 | 11.0 |
| **LASSO Cox** | 1.759 | 88.6 | 11.4 |
| **DeepSurv** | 1.434 | 83.5 | 16.5 |
| **Cox** | 20.718 | 85.2 | 14.8 |

**Notes:** Optimal cutpoints were determined separately for each model on its predicted risk score in the training set, using the maxstat method that maximizes the standardized log-rank statistic. The percentages of patients classified as low and high risk by each cutpoint are reported.

**Abbreviations:** GBSA, gradient boosted survival analysis; LASSO, least absolute shrinkage and selection operator; RSF, random survival forest.

**Table S9.** Comparison of the Extra Trees model with conventional clinical prognostic indicators on the test set.

| **Predictor** | **C-index (95% CI)** | **ΔC vs Extra Trees** |
| --- | --- | --- |
| **Extra Trees (machine learning)** | 0.843 (0.762–0.914) | Reference |
| TNM stage | 0.772 (0.659–0.873) | −0.071 |
| Ki-67 index | 0.705 (0.563–0.838) | −0.138 |
| Tumor grade | 0.699 (0.559–0.827) | −0.144 |

**Notes:** The Extra Trees model was compared with single-predictor Cox proportional hazards models fitted with each clinical prognostic indicator as the sole covariate on the test set. Harrell C-index and bootstrap 95% confidence intervals were computed for each model. ΔC denotes the difference in C-index relative to the Extra Trees model.

**Abbreviations:** CI, confidence interval; TNM, tumor-node-metastasis.

**Table S10.** Coefficients of the simplified nomogram (Cox proportional-hazards model on the five top-ranked variables) for predicting 3- and 5-year overall survival.

| **Variable** | **Coefficient (β)** |
| --- | --- |
| T stage (per unit) | 0.451 |
| Grade G2 (vs G1) | 1.724 |
| Grade G3 (vs G1) | 1.534 |
| Distant metastasis, M1 (vs M0) | 7.816 |
| TNM stage II (vs I) | −0.109 |
| TNM stage III (vs I) | 1.232 |
| TNM stage IV (vs I) | −6.577 |
| Ki-67 index (per %) | 0.024 |

**Notes:** The linear predictor is LP = Σ βᵢxᵢ, summing the coefficients above for a given patient. Predicted survival is S(t∣X) = S₀(t)^exp(LP), where the baseline survival is S₀(36 months) = 0.9939 and S₀(60 months) = 0.9892. Reference categories are T = 0, Grade G1, M0, and TNM stage I. Because distant metastasis (M1) and TNM stage IV are strongly collinear (stage IV is largely defined by the presence of distant metastasis), their individual coefficients are large and of opposite sign and should not be interpreted in isolation; their combined contribution for a metastatic stage IV patient is stable. This explicit formula pertains to the simplified nomogram only; the Extra Trees model is a non-parametric ensemble without a closed-form equation, and its predictions are provided through the SHAP analysis and the online calculator.

**Abbreviations:** LP, linear predictor; TNM, tumor–node–metastasis.

**Table S11.** External validation of the six survival models in the SEER cohort.

| **Model** | **C-index (95% CI)** | **AUC 1-yr** | **AUC 3-yr** | **AUC 5-yr** |
| --- | --- | --- | --- | --- |
| **DeepSurv** | 0.740 (0.736–0.744) | 0.807 | 0.790 | 0.777 |
| **RSF** | 0.728 (0.724–0.732) | 0.819 | 0.797 | 0.771 |
| **Extra Trees** | 0.719 (0.715–0.724) | 0.817 | 0.791 | 0.764 |
| **LASSO Cox** | 0.675 (0.671–0.680) | 0.769 | 0.740 | 0.713 |
| **GBSA** | 0.655 (0.651–0.660) | 0.738 | 0.718 | 0.688 |
| **Cox** | 0.600 (0.595–0.604) | 0.692 | 0.659 | 0.620 |
| **Wu et al. 2024 (variable set, refit)ᵃ** | 0.809 (0.805-0.812) | - | - | - |

ᵃFit and evaluated directly on the SEER cohort (in-sample), unlike the six primary models which were trained internally and externally validated on SEER; not directly comparable.

**Table S12.** External calibration of the Extra Trees model in the SEER cohort.

| **Time point** | **Calibration slope** | **ICI** | **E50** | **E90** |
| --- | --- | --- | --- | --- |
| **1 year** | 0.901 | 0.032 | 0.008 | 0.121 |
| **3 years** | 0.970 | 0.035 | 0.018 | 0.107 |
| **5 years** | 1.058 | 0.040 | 0.040 | 0.080 |

**Notes:** All six models were directly applied to the SEER cohort (n = 51,225) without further refitting or recalibration. Discrimination was assessed by Harrell C-index with 95% confidence intervals from bootstrap resampling and time-dependent AUC at 1, 3 and 5 years. External calibration of the Extra Trees model was assessed by cloglog-Cox calibration slope, integrated calibration index (ICI), and the 50th and 90th percentile of absolute prediction error (E50 and E90).

**Abbreviations:** AUC, area under the curve; CI, confidence interval; GBSA, gradient boosted survival analysis; ICI, integrated calibration index; LASSO, least absolute shrinkage and selection operator; RSF, random survival forest; SEER, Surveillance, Epidemiology, and End Results.

# **2.** Supplementary Figures

**Figure S1. Predictor inclusion and preprocessing workflow.**

**Figure S2.** Distribution of out-of-fold C-index from 5×5 nested cross-validation across six survival models.

**Figure S3.** Risk stratification across all six machine-learning models on the test set. Kaplan-Meier curves of overall survival in the test set stratified into high-risk and low-risk groups by each model's optimal cutpoint.

**Figure S4.** Decision curve analysis at 1 and 5 years.
